# Legitimate Wealth? How Wealthy Business Owners are Portrayed in the Press

**DOI:** 10.1007/s11211-022-00396-1

**Published:** 2022-09-09

**Authors:** Nora Waitkus, Stefan Wallaschek

**Affiliations:** 1 Department of Sociology, Tilburg University, Tilburg, Netherlands; 2 International Inequalities Institutes, London School of Economics, London, UK; 3 Interdisciplinary Centre for European Studies, Europa-Universität Flensburg, Flensburg, Germany

**Keywords:** Business owners, Elites, Wealth inequality, Legitimization, Frame analysis, Media, Germany

## Abstract

Germany has one of the highest levels of wealth concentration of any Western capitalist country. Research on the legitimization of economic inequality highlights that wealth elites tend to stress meritocratic arguments for legitimizing elite positions and wealth accumulation. However, whether this is also the case for wealthy business owners and how the media tends to portray those remains largely unknown. Drawing on a unique sample of 899 press articles from eight different media outlets between 2014 and 2018, we find a rather generous media debate. Based on descriptive evidence and a latent class analysis, we identify six latent frames illustrating how wealthy business owners are portrayed in the press. We show that the sources of wealth (inheritance, investment, entrepreneurship) are often used to highlight these owners’ deep economic relevance to the German economy, while the use of wealth is predominantly framed as a mean for profit-seeking. For wealthy business owners, moral evaluation of personal conduct is less present in the media and, when it is present, is rarely negative. Our study is the first analysis of press coverage of the wealthiest German business owners indicating a legitimizing media debate of high wealth concentration in an advanced capitalist society.

## Introduction

Economic inequalities are high and a persistent phenomenon in many countries worldwide (Alvaredo et al., 2018; Pfeffer & Waitkus, 2021; Piketty, 2020). In particular, the growing concentration of wealth among the world’s wealthiest one per cent has been identified as a key factor contributing to the increasing economic polarization of Western capitalist societies, giving rise to substantial societal and political problems (Piketty, 2014; Piketty & Zucman, 2014; Winters, 2011). During the past two decades, scholars’ growing interest in wealth concentration has revitalized sociological research on wealth elites and accumulation (e.g. Savage, 2015; Savage & Williams, 2008) as well as on the cultural processes that perpetuate and reproduce inequality (Kantola & Kuusela, 2019; Lamont et al., 2014; Massey, 2014; Smith Ochoa & Yildiz, 2022). In the latter vein, scholars have explored how different groups participate in the meaning-making processes of inequality by the “mobilization of shared categories and classification systems through which individuals perceive and make sense of their environment” (Lamont et al., 2014, p. 574).

In addressing the increasing concentration of wealth among those at the top of the distribution, sociologists have started to analyse the frames that help reproduce extreme inequality (e.g. Epp & Jennings, 2020; Grisold & Theine, 2017; Jaworski & Thurlow, 2017; Small et al., 2010). On the one hand, they look at how (wealth) elites and business owners themselves make sense of and legitimize their advantageous positions (Hecht, 2021; Kantola & Kuusela, 2019; Khan, 2011; Kuusela, 2018, 2020; Sherman, 2019). On the other hand, they explore how middle-class and economically disadvantaged groups are involved in creating shared meaning-making structures that underlie and drive inequality (for example Mijs, 2019; Prasad et al., 2009; Sachweh, 2012). Elites often explain increasing inequality by pointing to meritocratic ideals of hard work and persistence (Kantola & Kuusela, 2019), even as the “ordinariness” of elite lifestyles makes them seem more normal and middle-class than their fortunes indicate (Adamson & Johansson, 2020; Friedman & Reeves, 2020; Jaworski & Thurlow, 2017; Kantola & Kuusela, 2019). Studying different groups of actors and how they make sense of inequality is therefore hugely important to understanding why high levels of inequality can persist. This paper contributes to this scholarship from a different angle: by analysing media outlets and how they frame the concentration of wealth among business owners.

We argue that the press coverage of economic inequalities is of crucial importance for understanding the legitimacy of different inequality regimes (Grisold & Preston, 2020; Grisold & Theine, 2017; Piketty, 2020). Indeed, the press plays a substantial role in democratic societies by creating a public sphere that enables actors to communicate and exchange ideas, debate, deliberate, and inform (Wessler & Freudenthaler, 2018). Research shows that media debates on inequality tend to be rather one-sided, failing to link rising inequality to structural processes, personalizing the reasons why people face poverty, and naturalizing inequality by blaming “external” processes like economic globalization (Grisold & Theine, 2017, McGovern et al., 2020, Smith Ochoa, 2020). As scholars have also demonstrated, the media’s negative framing of deservingness and poverty tends to erode public support for welfare states while at the same time political actors seldomly address the topic of inequality in public debates (Diermeier & Niehues, 2021; Epp & Jennings, 2020; Smith Ochoa, 2020). Hence, how the media frames wealthy business owners remains an understudied topic, yet can illuminate the meaning-making processes behind persistenly high levels of inequality.

In this article, we are particularly interested in the frames—defined here as “an emphasis in salience of different aspects of a topic” (De Vreese 2005, p. 53)—that surround the (unequal) accumulation of wealth. How does the media frame sources of wealth, and does it morally evaluate owners and their families? How do owners employ wealth? We chose to focus our study on Germany, in particular, because of its comparatively high level of wealth concentration relative to other Western capitalist countries (Pfeffer & Waitkus, 2021)^1^ and because business owners and their families head the German Rich Lists (Hartmann, 2000).

As we show, media discussion of wealthy business owners in Germany remains largely tucked into frames surrounding entrepreneurialism, investment, and profit-seeking; to a lesser degree, the debate also circles around criticisms of inequalities and moral evaluations of personal conduct. After identifying these manifest frames in the text, we use a latent class analysis to detect additional latent frames in media coverage. In so doing, we underline our descriptive findings and demonstrate how the interconnectedness of these different frames in public debate bolster a broadly present legitimizing public discourse on wealth inequality in Germany.

In the rest of this paper, we review the literature on the legitimacy of wealth accumulation and inequality, give background information for the specific case of Germany and describe our data and methods, present the results of our descriptive statistics and latent class analysis, and finally summarize our findings and discuss the implications and limitations of our work in the conclusion.

## Legitimizing Wealth Accumulation and Inequality

Responding to rising economic inequalities, social scientists have recently renewed their focus on wealth elites overall (e.g. Korsnes et al., 2017; Savage & Williams, 2008) and business owners and corporate elites specifically (Adamson & Johansson, 2020; Mills, 2000; Moran, 2008). Nevertheless, wealthy business owners have attracted only limited attention from scholars—despite their leading position on Rich Lists (e.g. Korom et al., 2017) and increasing presence in the media in recent years (Littler, 2007). Alongside businesspeople’ rising public presence (Adamson & Johansson, 2020; Littler, 2007), since the 1990s economic inequality has taken on an increasing salience in the media. In turn, this has increased public concern about inequality (McCall, 2013), obliging business owners and other wealthy people to defend their vast fortunes (Friedman & Reeves, 2020; Moran, 2008). The “cultural means” (Adamson & Johansson, 2020) through which wealth concentration in the hands of the few is publicly legitimized requires more scrutiny, particularly because citizens tend to underestimate income levels (McCall, 2013; Mijs, 2019) and wealth inequality (Norton & Ariely, 2011; Norton et al., 2014). Studying publicly available information about wealth inequality and concentration, as well as how the media portrays that subject, are essential to understanding why people underestimate levels of inequality.

Sociological research has started to study various discursive elements such as frames (Small et al., 2010) in order to shed light on the inter-individual meaning-making process of inequality (Lamont et al., 2014; Massey et al., 2014). Below, we suggest that studies of legitimizing and framing wealth and inequality focus on three different sets of argumentation to explain the (deserving or not) wealth of individuals (McCall, 2013; Rowlingson & Connor, 2011). After giving an overview of existing studies on the sources of wealth, we discuss scholarly work on the usages of wealth, and finally review studies on the moral evaluation of personal conduct.

### Sources of Wealth

The literature on wealth and inheritance taxation suggests that the ***source of wealth*** is central when it comes to evaluating whether or not someone is deservingly rich (see also Rowlingson & Connor, 2011 for a similar categorization). Bastani and Waldenström (2021), for example, show that people become much more favourable towards redistributive policies when they know about the share of inherited wealth in society. Hence, if people believe wealth is mainly self-accumulated, they show stronger disapproval of wealth redistribution than if they see it as mere luck. In a similar vein, Sachweh and Eicher (2018) have shown for Germany that individuals tend to be less in favour of taxing high levels of wealth when they perceive this wealth to be the outcome of work rather than inheritance, marriage, or winning the lottery. Accordingly, the question of whether or not people are deservingly rich is closely linked to question how rich people have accumulated their wealth (i.a. merit, hard work, effort, and risk-taking Rowlingson & Connor, 2011; Sachweh, 2012). In addition, accumulating wealth through business success or entrepreneurial innovation can be seen as another meritocratic framing strategy vis-à-vis the “deservingly wealthy” (Bröckling, 2016; Kantola & Kuusela, 2019; Waitkus & Groh-Samberg, 2018). In contrast, undeserved—and therefore less legitimate—wealth is acquired through sources that are perceived as non-meritocratic, such as inheritance, speculation, or luck (Rowlingson & Connor, 2011; see also Kreidl, 2000). Hence, our first guiding frame for the coding of press articles is the source of wealth—whether this be an inheritance, returns on investments, or entrepreneurship and work.

### Use of Wealth

The literature on the legitimacy of richness and wealth further suggests that whether or not an individual’s wealth will be perceived by others as legitimate depends on the ***use of wealth.***^2^ For example, one’s existing wealth can be used to further the accumulation of capital, mainly in the form of capital returns on investments. Research has shown that this use of wealth may accompany more negative evaluations of profit-seeking (Ragusa, 2015; Rowlingson & Connor, 2011). The profit-seeking motive can also have a positive connotation—for example, when it is discussed in the context of job creation or investing in new technologies. This could be especially relevant to the German case, where the *Mittelstand* (midsized companies) has been framed as the backbone of the German social market economy (for example Mau, 2012; see more on this below).

Wealthy people further employ wealth in philanthropic activities to legitimize their riches. A study by Ragusa shows that people tend to be less enthusiastic about taxing inheritance when the wealthy use their fortunes for the common good. In contrast, they are much more in favour of taxation when they assume the wealthy use it for their own profits (Ragusa, 2015). Therefore, whether wealth is used for generating more profit (see above), is invested into the creation of jobs or goes into philanthropic activities matters, since philanthropic activities by foundations or wealthy individuals are largely presented as being for the common good (Glucksberg & Russell-Prywata, 2020; Sklair & Glucksberg, 2020).

Another way wealthy people use and employ the wealth is in the political sphere. Although wealthy business owners in Germany seldom run for office or use their wealth for political activities, they do  use party donations to indicate ideological alignment and political orientations, though to a much lower degree than in liberal economies (McMenamin, 2012). Nonetheless, they tend to favour centre-right parties (CDU/CSU and FDP, see also Polk, 2020). Although donations across the political spectrum have increased, family business owners in particular still stick to their political ideologies (Goerres & Höpner, 2014). This is relevant, as the policy preferences of the wealthy differ from those of the middle and working classes (Page et al., 2013), and politics tend to be more responsive to their interests (Bartels, 2016; Elsässer et al., 2021). In sum, our second frame focuses on the use of wealth and how the media covers the different ways of using one’s wealth: for sheer profit-seeking, philanthropic reasons, tax evasion, or for political lobbying purposes.

### The Moral Evaluation of Personal Conduct

A third set of arguments that we have identified in the literature focuses to the ***moral evaluation of the personal conduct*** of the wealthy (Hecht, 2021; Kantola & Kuusela, 2019; Lamont, 1992; Rowlingson & Connor, 2011; Sherman, 2019).^3^ Lifestyles and consumption patterns have always served as grounds for elite distinction and legitimization practices (Bourdieu, 1984; Elias, 1978; Veblen, 2017). In a recent study, Sherman finds that when it comes to consumption, wealthy New Yorkers define their expenditures as reasonable and basic, and draw a moral boundary between themselves and other elites whom they perceive to be materialistic, pretentious, greedy, or shallow (Sherman, 2018; see also Schimpfössl, 2018). Consumption choices, they imply, should indicate normality and ordinariness rather than the ostentatiousness that Veblen (2017) describes for the *nouveaux riches* (see also Bourdieu, 1984; Jaworski & Thurlow, 2017; Sherman, 2018). In the same vein, researchers find that wealthy business owners construct identities based on hard work, persistence, and normality in order to legitimize their privileged positions (Kuusela, 2018). Thus, in their study of female business owners’ autobiographies, Adamson and Johansson (2020) identify a downplaying of class and wealth differences as these owners stress their ordinariness and the universality of gender struggles. This self-portrait has been reinforced by TV media, who portray the wealthy as particularly resilient, hard-working, and psychological superior in comparison with ordinary people (Carr et al., 2021). Of course, this emphasis on ordinary lifestyles is a somewhat recent phenomenon (Friedman & Reeves, 2020). Over the past two centuries, the focus of elite lifestyles has shifted from aristocratic activities such as hunting, polo, and sailing (19th-century), to emerging “high” cultural forms such as theatre, art, ballet, and classical music (twentieth century), to today’s core interest in spending time with family, friends, and pets.

Friedmann and Reeves argue that this kind of lifestyle shift occurs when emergent groups challenge elites’ position in society. In times of severe economic inequality, the move towards an ordinary lifestyle is a reaction to popular attitudes, as “they [elites] face increasing suspicion from wider publics that they lack prosocial motives and, in turn, authenticity and moral character” (Friedman & Reeves, 2020, p. 325). By signalling the ordinariness of their lifestyles—through a focus on family orientation and simple hobbies—elites try to overcome this authenticity gap (ibid.).

Against this background, we would expect public debate to reflect on the personalities of these elites—to possibly highlight the strong family orientation among German business owners and/or to report either on their extremely lavish or (in the opposite vein) surprisingly ascetic consumer behaviour. In sum, our third frame focuses on how the media moral evaluates the personal conduct of our chosen study group, focusing on consumption patterns (is elite consumer behaviour restrained or ostentatious?), family dynamics, and general descriptions of the (moral) character of wealthy businesspeople.

Overall, we use these three lines of research on the legitimacy of wealth and inequality—sources of wealth, usages of wealth, and the moral evaluation of their personal conduct—as guiding themes for our empirical analysis of frames.^4^ The next section gives more context and information on our case and presents our analytic approach.

## Analytic Approach

This section discusses the details of our case study and the central role of (family) business owners in the German economy. We also present our sampling and coding strategies and our methods.

### Business Owners in the German Political Economy

The German capitalist market economy after World War II was mainly based on companies and family business embedded in multiple sets of rules and regulations between labour and capital interests (Streeck, 1997). Most companies were privately held, and only a small part of the productive capital was traded on the stock market (Dunlavy & Welskopp, 2007). Therefore, shareholding was highly concentrated and company shares seldom changed hands (Colli & Rose, 2008; Streeck, 1997, p. 241). These historical developments continue to shape the current economic structure in Germany, where the largest public and private companies are still associated with family businesses. Although business owners at the top of the German rich lists (and therefore also sit at the top of its wealth distribution), research on business owners mainly addresses intergenerational transmission and family ties (for example Berghoff, 2006; Stamm, 2016; Stamm et al., 2011) or the specific role of *Mittelstand*^5^ (family) business as the backbone of German capitalism (Berghoff, 2006; Kohl & Ergen, 2021; Streeck, 1997). Research on business owners and their wealth in Germany is particularly rare (Hartmann, 2000; Zellweger & Kammerlander, 2015); however, there is a strong association between the rise in wealth inequality since the 1990s and the growing business wealth at the top of the distribution (Albers et al., 2020).

### Sampling Strategy and Coding

To explore the frames for legitimacy, we use Manager Magazin’s annual German Rich List (similar to the Forbes list). We restrict our research to the five-year period (2014–2018) following the global recession—a time when the Eurozone crisis had nearly ended, but the COVID-19 pandemic had not yet begun. Further, we only study business owners who make it to the top ten more than once, which means (we assume) that they are publicly known. Following this strategy, we identify 16 business owners who either run their own businesses (the majority), or who co-own and run several companies in connection with one key brand (see overview in Table 1).

**Table 1:** The 16 wealthiest business owners in Germany (2014–2018)

*Family*	More than two generations	Two generations	Position last ranking (2018)	Net wealth € (in billions) 2018	Main company founded in (year)	Associated companies	Industry Branch
T. Albrecht	Yes	Yes	5	17.5	Aldi (1946)	Aldi Nord	Retail, Real Estate
K. Albrecht	Yes	Yes	4	21.8	Aldi (1946)	Aldi Süd	Retail, Real Estate
Herz	No	Yes	13	8.5	Tchibo (1949)	Mayfair, Vapiano, vorm. Tchibo	Shareholdings
Hopp	No	No	16	7.7	SAP (1972)	SAP, Dievini	Software, Shareholdings
Kühne	No	Yes	10	10.5	Kühne + Nagel (1890); Hapag-Lloyd (1970)	Kühne + Nagel, Hapag-Lloyd	Logistics, Shipping, Hotels
Liebherr	No	Yes	14	8	Liebherr (1949)	Liebherr-International	Mechanical engineering, household appliances, aviation technology, hotels
Oetker	Yes	Yes	14	8	Oetker (1891)	Oetker, Bankhaus Lampe, vorm. Hamburg Süd	Food, Bank, Hotels
Otto	Yes	Yes	8	13.5	Otto (1949)	Otto-Versand, ECE, Paramount, Park Property	Mail order, logistics, real estate, Shareholdings
Plattner	No	No	12	9.4	SAP (1972)	SAP	Software
Porsche	Yes	Yes	9	12	Porsche (1931); Volkswagen (1937)	Porsche, Volkswagen	Car industry, shareholdings
Quandt/Klatten	No	Yes	1	34	BMW (1916)	BMW, Altana, Delton, SGL Carbon	Car industry, shareholdings
Reimann	Yes	Yes	2	33	JAB Holding (1828)	JAB Holding; Keurig Dr Pepper, Coty	Beverages, cosmetics, veterinary clinics
Schaeffler	No	Yes	6	17	Schaeffler (1838); Continental (1871)	Schaeffler Technologies, Continental	Mechanical engineering, automotive supplier
Schwarz	No	No	3	25	Lidl (1973); Kaufland (1968)	Lidl, Kaufland	Retail, Real Estate
Thiele	No	No	7	15	Knorr-Bremse (1905); Vossloh (1888)	Knorr-Bremse, Vossloh	Automotive supplier, railroad technology
Würth	No	Yes	11	9.8	Würt (1945)	Würth-Gruppe	Tools trade

These companies are deeply enmeshed in Germany’s export-driven economy and strongly profit from economic globalization (Lane, 2000; Streeck, 1997). They include car companies and their suppliers (Quandt/Klatten, Schaeffler, Porsche, Thiele), supermarket chains and other food supply and production companies (Schwarz, K. Albrecht and T. Albrecht, Oetker, Reimann, Herz), as well as firms from other industries such as logistics (Kühne), tools (Würth), trade/services (Otto), and software (Hopp, Plattner). Some of them have profited massively from expropriation, forced labour, and/or supplying the Wehrmacht between 1933–1945 (Dean, 2008; Frei & Schanetzky, 2010; Windolf & Marx, 2022).^6^ While we are aware that not all of Germany’s wealth elite inherited their riches, only a small fraction of the families we studied had built up their own wealth (Schwarz, Thiele, Plattner, Hopp).

Based on this list, we searched the Factiva database for relevant articles, using the name of the family, affiliated companies, and either the German term “reich/Reichtum” (*rich*/*richness*) or “Vermögen” (*wealth*) in our keyword query.^7^ We selected a variety of media outlets, capturing different political–ideological orientations as well as print media types (see Table 4).

Following this methodology, we retrieved 899 articles for our research period.^8^ Our sample includes three daily quality newspapers (Süddeutsche Zeitung, Welt and Welt am Sonntag, taz), one economic daily newspaper (Handelsblatt), a weekly newspaper (Die Zeit), three weekly magazines (Spiegel, Stern, Focus), and a tabloid (Bild). This selection enabled us to draw the sample from various different types of media outlets with different distribution schedules (daily/weekly), all of which had a broad circulation and large readership (IVW, 2019).

*Second*, we developed our codebook alongside a pretest with a small sample of texts and that guides our analysis (see Appendix Tables 3 and 10). To test the viability of our initial themes and to identify new sub-themes, we developed an initial codebook using a deductive-inductive coding technique (see Table 3). We then revised this codebook, defining the coding categories to be used during the final coding of our sample. In total, we coded 641 (71.3%) of the 899 media articles selected (see Table 4). The number of coded articles varies by media outlet and family (see Table 5). For example, we only retrieved four articles for Liebherr, which is why we chose not to interpret the findings for this business owner. By contrast, our sample extensively covers Susanne Klatten and Stefan Quandt (main shareholders of BMW) or Wolfgang Porsche (a Porsche shareholder). The highest number of selected and coded articles in our sample came from the economic newspaper Handelsblatt and the liberal-left newspaper SZ, whereas less than half of them came from the tabloid press (Bild).^9^

### Methods

Our methodological approach to these articles was guided by frame analysis. Frame analysis is a technique that enables researchers to examine text in a structured and transparent manner (David & Baden, 2017; Entman, 1993; Snow, 2004). Framing means “to select some aspects of a perceived reality and make them more salient in a communicating text, in such a way as to promote a particular problem definition, causal interpretation, moral evaluation, and/or treatment recommendation for the item described” (Entman, 1993, p. 52). The process of selection and salience is crucial to understanding media coverage of certain issues and is used as a heuristic to grasp the meaning-making process in texts. Frames structure public debate by selecting and highlighting certain aspects of those debates, influencing how political and societal problems are perceived, and thus potentially mobilizing the public (Entman, 2007; Snow, 2004; Wallaschek, 2020). As De Vreese (2005, p. 52) notes, “frames may contribute to shaping social level processes such as political socialization, decision-making, and collective actions”. Which frames prevail in the media—and how they are presented—shapes people’s understanding of the issues, and influences their support or rejection of them (Ferrara et al., 2021).

To apply the frames approach to our study, we first manually coded manifest frames in the selected media articles. We then built on these findings by describing the manifest frames before applying latent class analysis (LCA). This method helped us to uncover latent frames in our data. As a probabilistic type of cluster analysis, LCA enables researchers to identify specific constellations for categorical data. It involves structural equation modelling and requires extensive interpretation of the results. Formally, one can obtain the best solution under this method when different test statistics—such as those produced by the Lo, Mendell, and Rubin test—are significant, and when the information criteria reach the minimum (Lo et al., 2001; Nylund et al., 2007; Vermunt & Magidson, 2004), although the result must also be theoretically meaningful. We used nine indicators for the LCA: (1) inequality, (2) source: inheritance, (3) source: investment, (4) source: entrepreneurship, (5) usage: philanthropy, (6) usage: profit, (7) moral: positive personal evaluation, (8) moral: negative personal evaluation, (9) moral: family orientation. We excluded less frequent references (i.e. those that appeared in less than 10% of all valid articles; see Table 2).

**Table 2:** Sources, use, and moral evaluation by owner (in %)

Owner			Sources of wealth			Use of wealth				Moral evaluation of personal conduct					
	#	%	%			%				%					
	Articles	Inequality	Inheritance	Investment	Entrepreneurship	Philanthropy	Profit	Politics/ Citizenship	Tax Evasion	Personality+	Personality−	Good capitalist	Family orientation	Ostentatious consump.	Restrained consumpt.
Quandt/Klatten	132	51	47	59	35	19	41	10	11	11	7	8	5	6	0
Porsche	90	10	18	57	37	12	11	2	2	12	37	0	37	1	3
Plattner	60	38	0	15	100	38	3	0	3	12	3	2	0	0	8
Hopp	45	20	2	44	98	27	9	0	0	16	4	4	2	2	2
Oetker	43	7	30	21	56	5	5	7	2	9	37	7	42	5	14
T. Albrecht	41	29	88	24	73	51	41	0	0	73	56	7	20	5	2
Otto	33	15	36	15	76	67	3	9	9	27	3	24	9	6	3
K. Albrecht	31	35	55	45	90	61	77	0	6	55	10	26	35	0	0
Kühne	28	36	39	79	86	54	57	39	36	50	25	18	4	32	0
Schaeffler	28	75	32	32	89	7	18	0	0	18	21	14	18	11	7
Reimann	26	46	46	50	92	12	27	0	15	19	8	0	27	12	0
Schwarz	25	60	8	40	72	28	24	12	4	28	16	0	4	0	0
Herz	24	0	67	67	42	25	21	4	13	25	0	13	25	8	0
Würth	20	90	5	30	100	45	60	0	15	60	5	15	30	30	5
Thiele	11	36	0	82	82	18	45	0	0	45	36	18	45	9	0
Liebherr	4	75	100	25	75	0	25	0	50	25	0	25	25	0	0
Total	641														

Multiple codes per articles are assigned, resulting in column percentages that do not add up to 100. Table sorted by highest position on the Rich List (see Table 1)

## Results

In this section, we will start by giving a quick overview of our results, focusing on the sources of wealth, use of wealth, and moral evaluation of personal conduct (see Table 2). We will then discuss our findings from the LCA.

### Descriptive Results

In general, the topic of inequality received scant attention in our media sample. About 35% of the sampled articles mention that the owner’s family in question is richer or wealthier than other families or individuals, either in Germany or around the globe. However, the number of references to inequality varies by family. For some families (Würth, Quandt/Klatten, Schaeffler, and Reimann), we find frequent references to this topic, whereas for others (Otto, Porsche, Oetker, Herz), it hardly makes news.

As we have already noted, how one describes the sources of a person’s wealth can legitimize or delegitimize its accumulation and the resultant inequality. People tend to accept the legitimacy of wealth if it is portrayed as the result of hard work, entrepreneurship, or merit—which is not the case if it comes from inheritance, luck, marriage, or investment (Rowlingson & Connor, 2011; Sachweh & Eicher, 2018). In our sample, most business owners are predominantly described as entrepreneurs; thus for eleven out of our 16 families, this angle appears more frequently than those of investment or inheritance. This is hardly surprising, given that the name of the company was one of our queried keywords. Nevertheless, while almost all the owners in our data have heirs in their ranks (see Table 1), this fact is only mentioned in about a third of all articles (Table 2). Investment as a source of wealth was referenced for all the business owners in our data, but more frequently for Kühne, Herz, Quandt/Klatten, Porsche and Thiele—the first four of which inherited large shares of their companies.

Regarding wealth usage, a quarter of our sampled articles mention philanthropic activities, with Otto scoring very high on this item (in 67% of the articles mentioning Michael Otto his philanthropic work in Hamburg, for example the co-financing of the Elbphilharmonie concert hall or a public art gallery, is noted), followed by Aldi-Süd (K. Albrecht) and Kühne. Another illustrative case of philanthropy depicted in the articles sampled was the Schwarz family foundation’s donation of 20 economics professorships to the Technical University Munich.

Philanthropic work was not relevant for other families (e.g. Reimann or Schaeffler), since they seem not engage (publicly) in such activities. By contrast, the profit-seeking motive comes up especially frequently in connection with the two Albrecht brothers, Thiele and Würth families. This may be due to their media depiction as success stories for new businesses, either during the German “Wirtschaftswunder” of the 1950s–60s  (in the cases of Albrecht and Würth) or following a series of economic challenges to their companies (in the case of Thiele). What is more, political engagement is hardly an issue in our sample. One exception is Kühne who is (alongside the state of Hamburg) one of the main shareholders of the German shipping company Hapag-Lloyd and has a financial engagement in the football club Hamburger SV. Otherwise, Quandt/Klatten are noticed when they donate to the German political parties CDU or FDP.

In contrast to our initial expectations, the sampled articles seldom mention tax evasion. We do find references to Kühne, Reimann, Quandt/Klatten, and Herz—owners who have transferred their business to their children in order to circumvent inheritance and capital taxation (Quandt/Klatten), or moved their headquarters to Switzerland, Austria, or Luxembourg to limit corporate taxes (Kühne, Reimann).

Table 2 also shows details on the moral evaluations of personal conduct that can be found in our sample. While the articles we selected presented relatively few such evaluations, articles that do include moral assessments appear slightly more inclined to give a positive evaluation in this regard (24% of all such articles) than a negative one (18%). While we did expect to find more positive characterization of companies as local *Mittelstand* firms (both of which are associated with being a “good capitalist”), we find this in only eight per cent of all articles, though this type of reference crops up more often for some (K. Albrecht, Otto, Schaeffler, and Thiele) than for others. The emphasis on family and family business was another element in the articles investigated (almost 18% of all articles). Here again, variation looms large and is particularly pronounced for K. Albrecht, Oetker, Porsche, and Thiele, with the first three experiencing tough and challenging intergenerational wealth- and power-transmission processes within their companies.

Lastly, consumption was less of an issue among the articles investigated. The few mentions we found on this topic focused on wealthy business owners’ extensive consumption and purchase of luxury goods, rather than their ascetic or restrained lifestyles. However, both consumption patterns receive predominantly positive framing. The articles portrayed high consumption as a personal reward for hard work, and low consumption as a signal of focused business ownership and a strict work ethic.

Overall, the descriptions show that the media coverage of the German business families in our sample rarely targets inequality, tends to discuss entrepreneurialism, has a slight tendency to view the personal conduct of those in this group as morally positive, and focuses much more on profit-seeking and philanthropy than on politics, taxes, and other issues. While no clear patterns emerge from these first descriptive results, we embark on a further task to uncover latent frames in the coded media articles.

### Latent Frames

The second step of our empirical analysis seeks to discover whether we can detect latent frames of press coverage on wealthy business owners. Using only indicators that occurred in more than ten per cent of our coded articles, we ran the LCA with nine indicators: (1) inequality, (2) source: inheritance, (3) source: investment, (4) source: entrepreneurship, (5) usage: philanthropy, (6) usage: profit, (7) moral: positive personal evaluation, (8) moral: negative personal evaluation, (9) moral: family orientation.

**Fig. 1:**
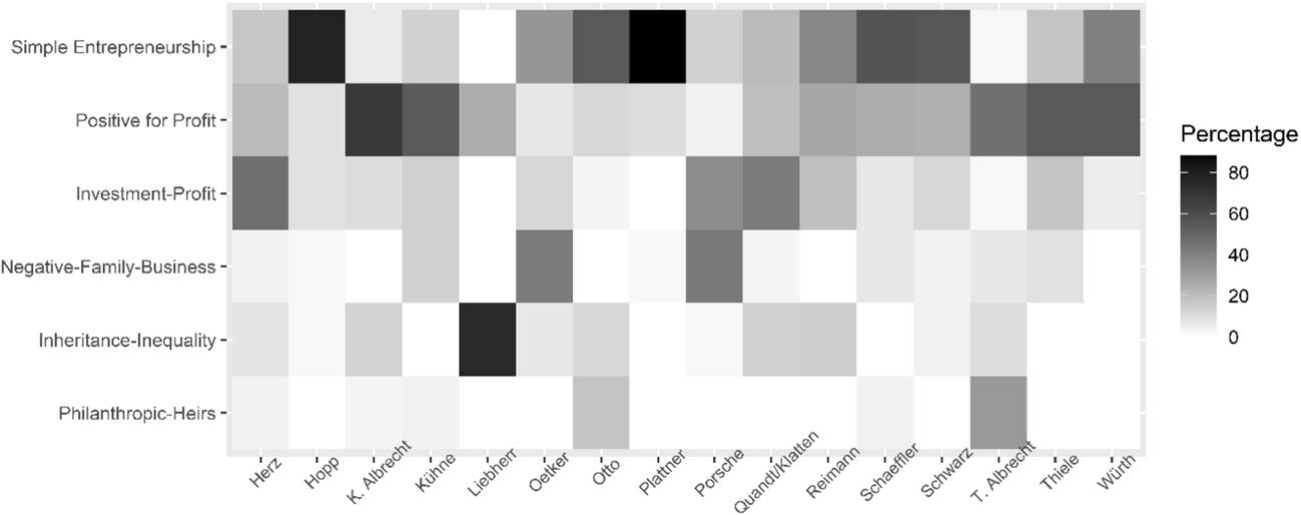
Heatmap of latent frames and wealthy business owners

After a thorough investigation of different latent class solutions (see Table 6 and 7), we opted for the six-class solution (as it yielded the most favourable test statistics and the most meaningful results). The six latent classes offer a preliminary focus on different aspects of these owners and can therefore illuminate which manifest codes go together (see Fig. 1). We start with the largest latent frame to be found in our sample:

About 35% of all articles in our sample simply cite entrepreneurship as the source of wealth, nearly ignoring all other topics. Therefore, we label this latent frame “Simple Entrepreneurship”. Investment as a source of wealth and owner philanthropy are scarcely covered, and the topic of inheritance is not addressed at all. This latent frame seems to be the most exemplary one for media coverage of wealthy business owners. The reports mainly focus on the companies and their economic development and prospects, with less coverage of the individual owners or their families, although their wealth is mentioned (but not inequality, for that matter). This frame is most often associated with Hopp and Plattner, founders of the SAP software company and predominantly seen as successful entrepreneurs “made in Germany”; however, we also see it in connection with Schaeffler and Schwarz. This economic success story as entrepreneurs seems to be the foundation to justify their wealth.

The second largest frame, which we call “Positive for Profit”, covers 22% of all the sampled articles. This frame highlights the unequal distribution of wealth and is much more concerned with wealth as the result of investment and entrepreneurship, rather than inheritance. While the philanthropy occasionally crops up, thematically the topics of profit orientation and positive personal evaluation predominate. K. Albrecht, Kühne, Thiele, and Würth families are associated with this latent frame. These three companies (retail, automotive supplier, and tools) represent the core of German capitalism (Streeck, 1997) and they established strong economic positions in Germany before expanding their businesses to other countries. Led over decades by men who are occasionally described (positively) as patriarchs and perceived as hard-working, these companies often anticipated what is economically sensible (for instance, by “inventing the retail discounter” in the case of Aldi-Süd [K. Albrecht]).

The third largest frame (found in 20% of all articles) especially focuses on investment and a for-profit orientation (“Investment Profit Frame”). The families most often associated with this frame are Quandt/Klatten, Herz, and Porsche. Even though these families represent the car industry sector (Porsche and BMW)—an economic cornerstone of German capitalism—media coverage on them strongly focuses on their new financial investment strategies and role as company shareholders (or, in the case of Herz, their investment in new companies, thus diversifying the company’s portfolio). Such reports highlight the profitability of these companies as well as the profit orientation of their owners. An interesting side note is that these owners all represent the bleaker part of German capitalism: the Quandt and Porsche families, in particular, have profited substantially from the Nazi regime—a historical fact, that is rarely mentioned in the press articles we analysed.

We label the fourth latent frame the “Negative Family Business Frame” (found in about 12% of our sample), since it includes articles that almost completely ignore the topic of inequality while describing all sources of wealth to an almost comparable amount. Noteworthy about this latent frame is its especially frequent use of negative evaluations of personal conduct and strong focus on family orientation—the strongest of all the frames we analysed. Owners associated with this sort of coverage are Porsche and Oetker, whose families made headlines regarding their family disputes.

By contrast, our fifth frame (the “Inheritance-Inequality Frame”) focuses on source of inheritance while still including some references to economic inequality. While only seven per cent of all articles fall into this category, we find that this frame tends to be more frequent when used in connection with Quandt/Klatten, Reimann, and K. Albrecht (Aldi-Süd). It is the only frame for which the topic of inequality is problematized and given crucial importance. For example, Quandt and Klatten were regularly covered by the press when the matriarch Johanna Quandt died and it was revealed that her BMW company shares had already been transferred to her children using tax loopholes which enabled her to (largely) circumvent inheritance taxation. Inheritance was a frequent topic after the founder of the retailer Aldi-Süd, Karl Albrecht, died in 2014. Both Albrecht brothers established foundations to control assets and avoid company breakup due to inheritance disputes. Furthermore, many obituaries in the media not only framed Aldi as positive German capitalism success story, but also emphasized the personal wealth Karl Albrecht accumulated through his entrepreneurial innovations.

Our last and smallest latent class is characterized by a strong emphasis on inheritance and philanthropy, as well as moral evaluations of personal conduct. Therefore, we label this latent frame “Philanthropic Heirs”. While the personal evaluations are predominantly positive, for this frame we also find more negative evaluations of these families than in other frames. Since only four per cent of all articles fall into this category, only T. Albrecht and Otto are worth mentioning. In this regard—as we have already mentioned—Otto receives particularly strong coverage for his philanthropic engagement in Hamburg.

Overall, we find six different latent frames that cover and link distinct aspects. While only two of six latent frames (Positive for Profit, Inheritance Inequality) regularly mention inequality, the largest frame we found is mainly concerned with a simple description of entrepreneurial activity. We assume that this is partially the result of our sampling strategy, since we specifically aimed to find articles on business owners and their companies and thus also retrieved a huge chunk of economic news articles. Another highlight of our latent class analysis is that personal moral evaluations are hardly observable but do have the tendency to evaluate these families in a positive way. This concerns consumption but also philanthropic activities, work ethics, and other positively connotated characteristics. Overall, we interpret these findings as constituting a rather legitimizing debate on wealth of German business owners.

## Conclusion and Outlook

This paper investigates media coverage and frames on wealthy business owners in Germany for the period 2014–2018. Drawing on a sample of 899 media articles, we investigate how the sources, use, and moral evaluation of personal conduct of wealthy business owners vary across owners and their associated families. Using latent class analysis, we further identify six latent frames of media coverage.

We find that wealthy business owners are mostly portrayed in a favourable and supportive way, with press coverage highlighting their entrepreneurial activities, investment and profit-seeking activities, and philanthropic engagement. This is exemplified by the latent class analysis, where only two out of the six frames we looked at assesses inequality in a critical way, and only one gives a negative evaluation of the personal conduct of those in our subject group. The media debate strongly focuses on these individuals and families in their role as (successful) business owners who have established and maintained their own companies, for which they also act as investors and shareholders.

Since the press articles presented here rarely link economic inequalities to the wealth accumulation of these business owners, we would argue that this coverage represents a rather legitimizing public portrayal of their wealth. In fact, a critical reporting on the richest business owners in Germany, which looks at the causes and consequences of wealth and economic inequality, hardly takes place in the media. This favourable double depiction of wealthy business owners in the media—in terms of both their economic actions and their personal conduct may be attributed to various factors. Of these, we would highlight three:

First, one could argue that the crucial historical role played by these business owners in the German economic system makes for less critical press coverage of them. For instance, the owners of the retailer Aldi (T. and K. Albrecht) or Lidl (Schwarz), the main shareholders of central companies in the German car industry (Quandt/Klatten for BMW, Porsche for Porsche and VW and Schaeffler for Continental) have shaped the picture of the successful German “rhine capitalism” after WWII, continue to promote Germany’s image as “world export champion,” and have been a key source of support for German *Mittelstand* companies with strong connections to the big companies along the supply chain.

Second—and along these same lines—there may well be a national bias in terms of coverage. It would be interesting to know whether journalists highlight more positive factors, such as their entrepreneurial activity and successful investments in their coverage of German wealthy businesspeople versus, for example, their coverage of US American tech company owners.

Consequently, a third possibility is that an internal media logic drives the media’s comparatively favourable depiction of these business owners: namely, journalists’ own need to access insider information and background knowledge. If critical coverage might cause them to lose access to these insider sources, then their own professional closeness with their subjects may well prevent journalists from taking a more critical stance. A more detailed analysis of the relationship between journalists and their media coverage of business owners is thus a promising avenue for future research.

Whatever the reason for this overall fairly uncritical assessment of German wealthy business owners, it appears to reflect a broader legitimizing perspective on the capitalist system as a whole. Even when the articles we analysed mentioned wealth concentration and inequality, they did not systematically question the capitalist system or economic inequality in particular. This finding falls in line with previous research (Grisold & Theine, 2017; Jaworski & Thurlow, 2017; Schneider et al., 2017; Smith Ochoa, 2020). In this sense, our conclusion—that the media legitimizes the wealth of German business owners by positively depicting the terms of their entrepreneurship, investments, and economic success—contributes to a larger and growing debate over media coverage of economic inequality.

Our study comes with some limitations. Since our efforts to study the coverage of German business owner remain descriptive, we cannot evaluate whether these framing and reporting activities have shaped how the public perceives inequality. To explore whether and how press coverage of business owners may shape public discourse and public political orientations, particularly with regard to inequality, by looking at media and survey studies in connection with each other (Epp & Jennings, 2020).

Second, our period of study is right between two major economic crises—after the global recession and the Eurozone crisis and before start of the Covid-19 pandemic in 2020. In times of economic crisis, we would expect to find more extensive debate on inequality and economic disparities (as it has been shown by McArthur & Reeves, 2019; Schneider et al., 2017; Smith Ochoa, 2020). Possibly, the journalistic focus on entrepreneurial activities was a function of our defined research period, during which no major crisis dominated the media coverage. Future studies could approach this in one of two ways. Either they could look at the impact of global crises on the framing of wealth inequalities, or they could tackle the issue from a more long-term perspective, tracking changes in public discussions of inequality over decades or even centuries. In so doing, scholars could explore how the press helps to maintain current inequality regimes by offering more systematic accounts of how the media shape discourses on inequality over time (such as McCall, 2013; McGovern et al., 2020).

Our decision to focus exclusively on Germany is another limitation of this study. As we have argued above, the role of German business owners—mostly family business owners—is quite unique internationally; however, how Germany business owners at the top of the wealth distribution compare to other country’s top wealth holders remains to be studied. For example, since in the US self-made tech billionaires occupy the top ranks of the Forbes list, a comparative study on the framing of (wealth) inequality in different contexts could illuminate the degree to which far country-specific accumulation and legitimization strategies vary. It would be particularly interesting to know whether German business owners are portrayed more favourably than American business owners, for example, who are also covered extensively in the German press. Third, future research should investigate the media coverage of wealthy business owners who profited from the Nazi regime. Among the 16 families we analysed for this study, eight seem to have economic as well as interpersonal connections with that regime. Their activities in this regard ranged from the exploitation of forced labourers (e.g. Quandt, Reimann), supplying the Wehrmacht (e.g. Schaeffler) to running the logistics for deportations across Europe (e.g. Kühne + Nagel). While historians have extensively documented the role of business elites in the Nazi economy (e.g. Dean, 2008; Frei & Schanetzky, 2010; Tooze, 2006; Windolf & Marx, 2022), the impact of this historical plunder on current levels and perceptions of wealth inequality remains a research gap worth addressing. Since this topic rarely came up in our sample, we had to refrain from analysing it further (Becker & Waitkus, 2021).

To conclude, this study emphasizes the scholarly relevance of meaning-making processes of inequality (Lamont et al., 2014) that take place in the media. Through our analysis of press articles on wealthy business owners in Germany, we argue that extreme economic inequality can be maintained and reproduced through channels that are generally overlooked by stratification scholars. It may well be that legitimization of inequality is not simply a matter of individual or collective belief systems; it may also be driven by publicly available knowledge in the form of press coverage of wealthy business owners. Given that the articles we studied rarely tackle inequality, inheritance, and other more critical sets of arguments regarding the wealthy, one might argue that these legitimatizing frames result in a lack of publicly available knowledge about the “true” distribution of wealth and its profiteers, thereby upholding vast levels of wealth inequality in Germany.

## Figures and Tables

**Table 7:**
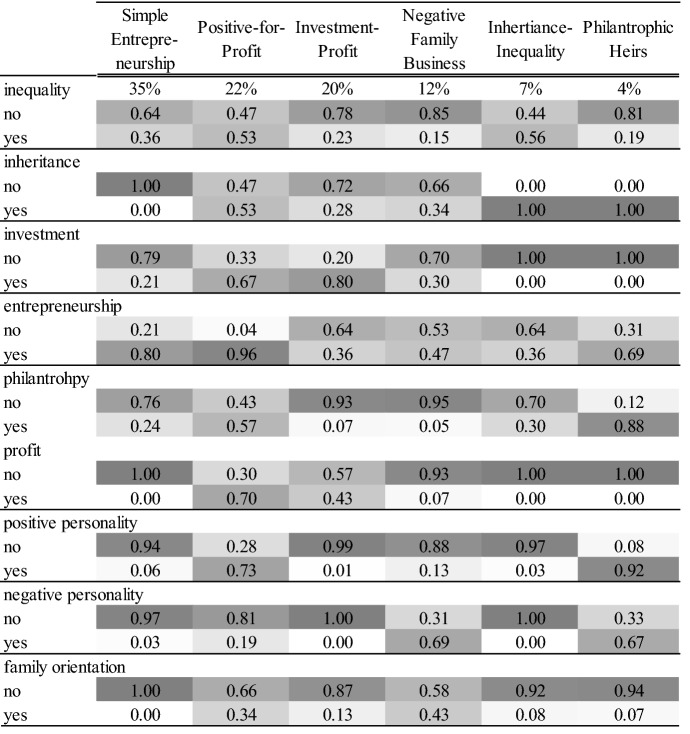
Characterization of LCA

## Appendix A: Tables

See Tables

**Table 3:** Coding scheme

Meta data	Newspaper	Inequality an issue	Sources of wealth	Usage of wealth	Moral evaluation of personal conduct
Year	Source section: politics, news, general issues	Inequality regarding wealth, poverty, tax mentioned?	Wealth as inheritance	Philanthropy	Personal moral evaluation, positive
Source	Source section: business, economy		Wealth as investment	Profit	Personal moral evaluation: negative
Text title	Source section: boulevard, culture, feuilleton		Wealth as entrepreneurship	Tax evasion	The good capitalist
Family				Politics citizenship	Family orientation
					Restrained consumption
					Ostentatious consumption

3,

**Table 4:** Overview media outlets

Media outlet	Publication frequency	Type of outlet	Sampled articles		Used articles	
			Number	Per cent of total (%)	Number	Per cent of sampled articles (%)
Handelsblatt	Daily	Newspaper	275	30.59	204	74.18
Die Welt (incl. WaS)	Daily	Newspaper	175	19.47	126	72.00
Sueddeutsche Zeitung (SZ)	Daily	Newspaper	206	22.91	167	81.07
Die tageszeitung	Daily	Newspaper	46	5.12	29	63.04
Bild	Daily	Tabloid	78	8.68	34	43.59
Zeit	Weekly	Newspaper	10	1.11	9	90.00
Der Spiegel	Weekly	Magazine	50	5.56	35	70.00
Focus	Weekly	Magazine	34	3.78	21	61.76
Stern	Weekly	Magazine	25	2.78	16	64.00
Total	–	–	899	100	641	70.71

^4^,

**Table 5:** Valid articles per owner and section in newspaper

	Sample *N*	Valid *N*	Section (in percentage)			Total
			Politics	Economy	Culture	
T. Albrecht	53	41	37	51	12	100
K. Albrecht	57	31	42	48	10	100
Herz	33	24	42	58	0	100
Hopp	47	45	20	71	9	100
Kühne	54	28	39	43	18	100
Liebherr	4	4	75	25	0	100
Oetker	92	43	26	60	14	100
Otto	62	33	52	39	9	100
Plattner	67	60	20	67	13	100
Porsche	121	90	23	69	8	100
Quandt/Klatten	140	132	27	67	6	100
Reimann	28	26	0	100	0	100
Schaeffler	37	28	32	64	4	100
Schwarz	59	25	20	80	0	100
Thiele	13	11	18	82	0	100
Würth	32	20	20	20	60	100
Total	899	641	27	63	10	100

^5^,

**Table 6:** Model fit statistics LCA

Classes	# of free parameters	Loglikelihood	AIC	BIC	Adj. BIC	Entropy	VLMR Test	LMR adj. LRT Test	H0 vs H1	AvLCP	Size
2	19	− 3239.592	6517.185	6601.982	6541.659	0.760	0.000	0.000	0.000	0.948–0.903	151 (24%)/490 (76%)
3	29	− 3201.794	6461.588	6591.016	6498.943	0.628	0.766	0.768	0.000	0.903–0.762	158 (25%)/161 (25%)/322 (50%)
4	39	− 3162.966	6403.932	6577.99	6454.167	0.630	0.037	0.038	0.000	0.868–0.718	115 (18%)/181 (28%)/144 (23%)/201 (31%)
5	49	− 3136.288	6370.576	6589.265	6433.693	0.704	0.071	0.073	0.000	0.873–0.741	249 (38%) 146 (23%)/77 (12%)/146 (23%)/23 (4%)
6	59	− 3117.934	6353.869	6617.187	6429.866	0.759	0.000	0.000	0.000	0.886–0.798	75 (12%)/145 (22%)/222 (35%)/23 (4%)/47 (7%)/129 (20%)
7	69	− 3105.885	6349.77	6657.719	6438.648	0.767	0.235	0.239	0.040	0.900–0.763	179 (28%)/23 (4%)/73 (11%)/47 (7%)/133 (21%)/113 (17%)/73 (11%) 8

^6^, 7,

**Table 8:** Frames and media outlets

Frame	Bild	Focus	Handelsblatt	SZ	Spiegel	Stern	Die Welt (incl. WaS)	Zeit	taz	Total
Simple Entrepreneurship	6	3	30	26	5	1	23	2	5	100
Positive for Profit	7	3	27	27	6	5	17	1	8	100
Investment Profit	4	4	30	26	9	3	19	2	3	100
Negative Family Business	5	4	36	31	5	1	15	0	3	100
Inheritance Inequality	4	2	45	19	0	2	23	0	4	100
Philanthropic Heirs	0	4	52	22	0	4	17	0	0	100
Total	5	3	32	26	5	3	20	1	5	100

^8^, and

**Table 9:** Owner occurrence per media outlet

	Bild	Focus	Handelsblatt	SZ	Spiegel	Stern	Die Welt (incl. WaS)	Zeit	taz	Total
T. Albrecht	2.4	2.4	36.6	24.4	7.3	4.9	17.1	2.4	2.4	100
K. Albrecht	6.5	0.0	35.5	29.0	0.0	0.0	16.1	3.2	9.7	100
Herz	4.2	0.0	50.0	8.3	0.0	0.0	37.5	0.0	0.0	100
Hopp	8.9	2.2	44.4	17.8	8.9	0.0	13.3	2.2	2.2	100
Kühne	0.0	0.0	21.4	10.7	3.6	3.6	28.6	0.0	32.1	100
Liebherr	50.0	0.0	50.0	0.0	0.0	0.0	0.0	0.0	0.0	100
Oetker	7.0	4.7	34.9	30.2	4.7	0.0	14.0	0.0	4.7	100
Otto	3.0	3.0	36.4	12.1	0.0	9.1	27.3	0.0	9.1	100
Plattner	6.7	8.3	30.0	18.3	11.7	1.7	18.3	1.7	3.3	100
Porsche	4.4	8.9	26.7	32.2	6.7	1.1	17.8	0.0	2.2	100
Quandt/Klatten	4.6	1.5	27.3	35.6	6.1	3.8	14.4	3.0	3.8	100
Reimann	0.0	3.9	57.7	7.7	3.9	0.0	26.9	0.0	0.0	100
Schaeffler	10.7	0.0	17.9	28.6	3.6	7.1	32.1	0.0	0.0	100
Schwarz	0.0	0.0	12.0	32.0	8.0	0.0	40.0	4.0	4.0	100
Thiele	0.0	0.0	36.4	54.6	0.0	0.0	9.1	0.0	0.0	100
Würth	15.0	0.0	30.0	35.0	0.0	5.0	15.0	0.0	0.0	100
Total	5.3	3.3	31.8	26.1	5.5	2.5	19.7	1.4	4.5	100

^9^.

## Appendix B: Codebook

### Anonymized Version

The unit of analysis is the article in the newspaper or magazine. All variables are “1” if this category applies to the article, and “0” if this does not apply and (except for the meta data) (Table 10).

**Table 10:** Categories

Meta data	Newspaper	Inequality framing	Legitimacy		
			Source of wealth	Use of wealth	Moral evaluation of personal conduct
1. No	6. Section. Politics, General Interest, general News	9. Inequality mentioned?	12. Source: Inheritance	15. Philanthropy	19. Personal moral evaluation: positive
2. Year	7. Section: Economics		13. Source: Investment?	16. Profit	20. Personal moral evaluation: negative
3. Paper	8. Section: Culture, Feuilleton, Boulevard		14. Source: Entrepreneurship	17. Tax Evasion	21. “The good capitalist”
4. Title				18. Politics, Citizenship	22. Family orientation
5. Owner					23. Restrained consumption
					24. Ostentatious consumption

#### Meta Data

*Year*—Year of Publication

*Paper*—Name of Newspaper/magazine

*Title*—Title of the article

*Family*—Owner’s family name.

#### Newspaper

*Section: Politics, general news, local news* (0/1)—self-description by the newspaper/magazine

Section: *Economics* (0/1)—(s.a.)

*Section: Culture, Feuilleton, Boulevard* (0/1)—(s.a.).

*Inequality framing* (0/1)—Inequality (Poverty, Richness, Wealth is mentioned in an article). This is defined as a relation, meaning it is not sufficient to mention on the Forbes List but as a relation towards another group, society as whole, or other countries. For example, “Klatten is a rich woman” would be coded as 0, “Klatten is richer than x” or “the richest” would be coded as 1.

#### Source of Wealth (Multiple Entries Possible)

*Source of Wealth: Inheritance* (0/1)—Inheritance is mentioned as source of the owner’s family or individuals’ wealth. Terms like heir, heiress, etc., care being used.

*Source of Wealth: Investment* (0/1)—Investments into companies, etc., are mentioned as one source of wealth. Words such as investors, share- or stockholders are being used.

*Source of Wealth: Entrepreneurship* (0/1)—are business activities mentioned as a source of wealth? Descriptions such a company, industrial family, founder of X, owner of X are being used.

#### Use of Wealth (multiple Entries Possible)

*Philanthropy/Orientation towards the common good* (0/1)—Mentioning of charities, philanthropies, cultural or social engagement.

*Tax avoidance* (0/1)—Wealth and wealth accumulation is associated with tax evasion. For example, it is mentioned that the use of charities facilitated tax optimization or is described as the circumvention of inheritance taxation.

*Profit orientation* (0/1)—The wealth of the owners and his family is described and used as investment, for purchasing other companies etc.

*Politics /Citizenship* (0/1)—The owner or his family is assigned a particular influential role in the political sphere or as citizens. For example, party donations, being friends with politicians or other influential people that are mentioned in the article.

#### Moral Evaluation of the Personal Conduct (Multiple Entries Possible)

*Personal evaluation positive* (0/1)—Are moral evaluation being used to describe the owner? Particularly positive connotated adjectives such assertive, precise, ambitious, generous, engaged, and strong.

*Personal evaluations negative* (0/1)—Are moral evaluations used to describe the owner? Particularly negative connotated adjectives such pedantic, selfish/self-serving, annoying, climbing over dead bodies (“geht über Leichen”), etc.

*“The good capitalist”* (0/1)—The article mentions job creation, economic growth, being an exemplary employer (e.g. caring about the work-life balance of the employees), caring employer or regional anchoring of the company are mentioned or being described as part of the *Mittelstand.*

*Family orientation* (0/1)—The family is described as a hoard of stability, family ties are emphasized, etc. Family as the backbone of the company. The kinship between children and over generations is emphasized, the children are mentioned as being groomed into the family business, etc. (Note: only mentioning of other family member’s names is not sufficient).

*Restrained Consumption* (0/1)—The article mentions an ascetic or restrained lifestyle, including simple consumption choices, etc. (no visibility of wealth, cars, jewellery, etc. (i.e. only buys at discounters or in cheap clothing stores; example from the material: “he only buys [his clothes] at C&A”).

*Ostentatious Consumption* (0/1)—The article mentions the excessive and luxury consumption, visibility of cars, jewellery houses, etc.

## Footnotes

1 The top 10% in Germany hold 60% of all net wealth, which makes Germany the country with the fifth highest wealth concentration among the 28 countries investigated in a recent OECD report (Balestra und Tonkin 2018, p. 15).

2 Rowlingson and Connor (2011) call these sets of arguments incentives for wealth creation.

3 As Sherman notes, the debate on the worthy and unworthy rich (or the 'good' and the 'bad' rich) detracts attention away from discussions about the unequal distribution of wealth (Sherman, 2019).

4 Although we differentiate them analytically, we are well aware that for example philanthropy can be both a source and a usage of wealth. A similar argument can be made with regards to lobbying and tax evasion. We thank an anonymous reviewer for this comment.

5 The term *Mittelstand* refers to small and medium sized companies with less than 500 employees and an annual turnover of less than 50 million € (IfM Bonn, 2019). Historically, the term referred to self-employed small business owners in craft, industry and commerce (Mau, 2012). Anecdotally, the very wealthy like to describe their business as being part of the *Mittelstand* as well, though this is more of a moral evaluation than an actual description, given the size of their companies and wealth.

6 We cannot investigate this issue systematically in this paper, but we do address it in our conclusion as an avenue for future research.

7 For instance, we used the following keyword list to capture Susanne Klatten and Stefan Quandt (main shareholders of BMW) in press articles: '(Quandt and BMW and reich) or (Klatten and BMW and Reichtum*) or (Quandt and BMW and Vermögen*) or (Klatten and BMW and Vermögen*)'.

8 While applying this procedure increased the likelihood that we would find the most relevant articles for this study, we still retrieved 'false positives' that just briefly mentioned the family and company name whilst reporting about wealth issues in other countries. This happened especially frequently with Bild. We further excluded article duplicates published in different (regional) versions of the same newspaper, as well as short articles (like death notices, or calendars with regional cultural events).

9 This shows that the media outlets covering news of wealthy business owners and their actions as (co-)owners of companies are predominantly top-quality ones. These outlets focus more on ‘hard news,’ while tabloid presses include more ‘soft news’ in their reportage (Reinemann et al., 2012). However, if we had searched for media articles by company name might have biased our sample towards more economically-focused publications (such as Handelsblatt) as opposed to tabloids (such as Bild). We will discuss this in our conclusion.
